# Ambient-Stable mRNA Medicines: Emerging Paradigms in Dry and Solid-State Formulation

**DOI:** 10.3390/ph19030370

**Published:** 2026-02-26

**Authors:** Mohamed El-Tanani, Syed Arman Rabbani, Adil Farooq Wali, Frezah Muhana, Alaa A. A. Aljabali, Yahia El-Tanani, Rakesh Kumar

**Affiliations:** 1 RAK College of Pharmacy, Ras Al Khaimah Medical and Health Sciences University, Ras Al Khaimah 11172, United Arab Emirates; arman@rakmhsu.ac.ae; 2 Princess Sarvath Community College, Amman 11196, Jordan; f.muhana@ammanu.edu.jo; 3 Department of Pharmaceutics and Pharmaceutical Technology, Faculty of Pharmacy, Yarmouk University, Irbid 21163, Jordan; alaaj@yu.edu.jo; 4 Royal Cornwall Hospital Trust, NHS, Truro TR1 3LJ, UK; y.el-tanani@nhs.net; 5 Amity Institute of Pharmacy, Amity University, Panchgaon, Gurgaon 122412, India; rakeshk.bhardwaj98@gmail.com; 6 Department of Pharmacy, Jagannath University, Bahadurgarh 124507, India

**Keywords:** mRNA therapeutics, ambient stability, solid-state formulation, lyophilization, lipid nanoparticles, predictive design

## Abstract

The medical field now uses mRNA therapeutics to deliver fast programmable treatment options through versatile vaccination platforms. The worldwide adoption of mRNA therapeutics faces a major obstacle because these molecules require extreme cold storage and transportation systems. mRNA stability establishes a fundamental scientific and industrial challenge which requires researchers to unite formulation design with process control and material engineering for cold-chain independence. Current knowledge about RNA hydrolysis and lipid oxidation and water-mediated degradation is combined with new methods for solid-state stabilization through lyophilization and spray-freeze-drying and thin-film technologies. Mechanism such as vitrification, water replacement and excipient RNA interactions are assessed to establish the fundamental chemical properties needed for extended product stability. Advanced mRNA development strategies are also examined, including self-amplifying and circular RNA structures and nano-glass and metal–organic frameworks and artificial intelligence-based predictive design for creating stable mRNA formulations at room temperature. This review examines manufacturing and regulatory and logistical obstacles which affect real-world implementation of mRNA therapeutics through assessments of production scale and product quality tests and packaging strength and tropical environment testing. The combination of research findings presents a path to develop mRNA medicines which maintains their effectiveness when stored at 25 °C or above, thus enabling worldwide access to RNA-based treatments. The development of mRNA into a durable therapeutic platform requires scientists to merge molecular research with process development and regulatory standardization.

## 1. Introduction

The medical field now uses mRNA technology to create vaccines and treatments which generate specific biological responses through programmable mechanisms [1]. The fast design process and large-scale production capabilities and strong immune response properties have made mRNA the leading therapy for infectious diseases and cancer treatment and rare genetic disorder management [1,2]. The worldwide success of mRNA vaccines against COVID-19 proved that nucleic acid-based medicines could move from design to clinical use within months instead of years [3]. The successful deployment of mRNA vaccines against COVID-19 revealed a major weakness that blocks wider medical applications of mRNA technology because mRNA molecules degrade easily [4].

The chemical structure of mRNA makes it more prone to degradation than DNA. The single-stranded structure of mRNA together with its reactive ribose backbone makes it highly vulnerable to hydrolytic and oxidative degradation which results in structural breakdown when exposed to body fluids or environmental conditions [5]. The current mRNA-based vaccines and treatments require storage at sub-zero temperatures between −20 °C and −80 °C as they lose their effectiveness when exposed to heat [6]. The storage requirements for mRNA-based products create substantial operational and financial challenges as they need specialized freezers and temperature monitoring systems and continuous dry ice or liquid nitrogen supply [6]. The storage requirements for mRNA-based products create significant challenges for low- and middle-income countries because these nations lack stable power grids and proper refrigerated transportation systems [7]. The COVID-19 pandemic revealed how vaccine and therapeutic access depended more on delivery systems than scientific capabilities [7].

The degradation of mRNA occurs through various dependent pathways which affect its stability at the molecular level. The ribose 2′-hydroxyl group in mRNA triggers intramolecular transesterification reactions that break down the phosphodiester backbone, and guanine and adenine bases become modified through oxidative reactions when pH levels rise or metal ions appear [5]. The protection of mRNA from nucleases and improved cellular entry becomes possible through its encapsulation within lipid nanoparticles (LNPs) [8]. The stability of LNP carriers faces challenges because ionizable lipids tend to degrade through oxidation, and hydrolysis and freeze–thaw cycles lead to phase separation and RNA release [9]. The storage of mRNA at low temperatures slows down degradation reactions but does not prevent them from happening while making production and distribution operations more complicated [6].

The requirement for cold-chain infrastructure has become the primary barrier which prevents mRNA therapeutics from advancing. The requirement for cold storage increases production costs and generates additional greenhouse gas emissions through refrigerated transportation while creating therapy access inequalities between areas with cooling systems and those without [7]. The development of mRNA technology for chronic disease treatment requires researchers to create stable formulations which maintain their effectiveness under normal and room-temperature conditions [10]. The achievement of clinical and commercial success depends on these applications requiring storage stability for multiple years and fast reconstitution and consistent product potency.

The study of mRNA formulation stability remains a critical matter, although scientists have not given it sufficient attention. Scientists dedicate most of their research to mRNA sequence optimization and immunogenicity reduction and delivery system development, but they spend little time studying physical degradation processes and stabilization techniques [4,8]. The current stability research investigates vaccine formulations, but therapeutic applications need different storage conditions because they must endure for extended periods and multiple dosages. The scientific community depends on mRNA stability engineering to advance their research while organizations can use this field to develop their operations [9,10].

This review shows that mRNA medical treatments need formulation stability as their basic requirement for stability. This study investigates mRNA degradation processes through laboratory tests while it explains stability factors present in both liquid and solid forms and assesses new methods for developing heat-resistant mRNA preparations. This review evaluates all essential factors which enable laboratory discoveries to become available sustainable medical treatments for patients. It combines information from chemistry, materials science, pharmaceutical engineering and public health to establish a method for creating mRNA medications which maintains their effectiveness when stored at room temperature. Finally, this review establishes a theoretical framework to create mRNA medicines which maintain their effectiveness under normal temperature conditions for worldwide distribution of RNA-based therapeutic products.

The therapeutic class of mRNA contains various molecular modalities which differ structurally and functionally, as these differences affect both stability and formulation requirements and storage needs. The two methods produce different results which become essential when vaccine stability requirements exceed the needs of vaccines that require cold storage [1,4].

The present vaccine platforms depend on conventional linear mRNA which exists as a single-stranded molecule with a cap and polyadenylated structure that makes it susceptible to hydrolytic backbone cleavage and oxidative base modification [5,10,11]. The process of productive translation needs complete transcript preservation, so any form of transcript shortening or chemical alteration will cause significant loss of potency [11,12]. The process of linear mRNA stabilization requires scientists to preserve exact moisture levels and molecular binding conditions while using particular rehydration methods when storing materials under solid-state conditions [13,14].

**Figure 1:**
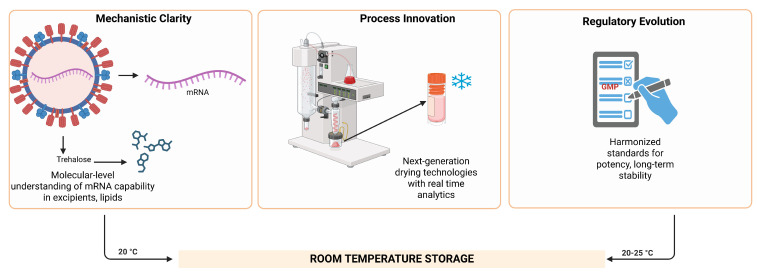
Modality-specific stability determinants for mRNA therapeutics. This figure illustrates structural differences among linear mRNA, self-amplifying RNA (saRNA), and circular RNA (circRNA), highlighting their respective vulnerabilities to hydrolysis, oxidative degradation, and shear stress. Key formulation implications such as excipient selection, drying kinetics, and encapsulation efficiency are mapped to each modality. The schematic emphasizes how molecular architecture drives stability requirements for ambient storage. Created in BioRender. Wali, A.F. (2026) https://BioRender.com/56k0ply, accessed on 2 February 2026.

The process of saRNA formulation becomes more complex because of its distinctive properties. The saRNA encoding viral replicase enables antigen-encoding RNA amplification within cells which results in needing only small amounts of the vaccine for administration [15]. The large molecular structure of this compound makes it more prone to break down when subjected to shear forces and also causes problems with complete encapsulation and formulation and drying process uniformity [16,17]. The specific requirements of these characteristics restrict the selection of excipients and drying processes and solid-matrix uniformity when storage under normal conditions is the goal [18,19] (Figure 1).

Circular RNA (circRNA) has emerged as an alternative modality with enhanced intrinsic resistance to exonuclease-mediated degradation due to the absence of free 5′ and 3′ termini [11]. The design structure of this feature maintains chemical stability, but circRNA encounters particular challenges during translation and formulation steps because it requires internal ribosome entry sites for translation and demonstrates variable protein production and lacks approved medical uses [4,20]. The circularization process defends against specific degradation mechanisms, yet scientists need to address three main issues: water content management, fat crystal behavior and protein structure flexibility [20,21].

The molecular structure of the material needs to meet specific stability criteria which stem from its planned medical use. The storage needs for prophylactic vaccines provide some flexibility, but therapeutic mRNA products used for chronic treatment require long-term stability maintenance for multiple years, and they need to demonstrate consistent reconstitution performance and protein production stability [2,7]. Researchers need to develop stability definitions which fit their particular storage systems and research goals that extend past conventional RNA stability assessments [9,10].

The identification of these differences becomes essential for developing logical mRNA medicine formulations and for establishing realistic stability targets for mRNA drugs which can remain stable at room temperature. The development of stabilization methods needs to consider three essential factors: delivery system requirements, manufacturing limitations, and the specific molecular characteristics and therapeutic goals of mRNA products [8,12].

**Figure 2:**
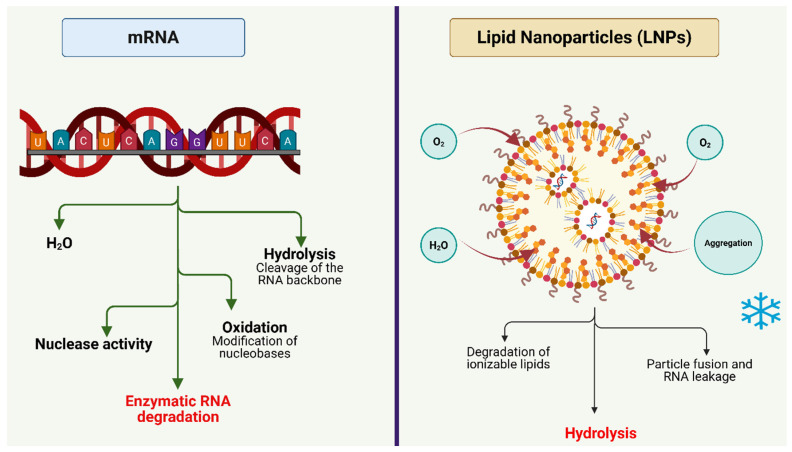
Design drivers linking molecular modality to solid-state stabilization targets. A conceptual framework showing how RNA size, secondary structure, and intended clinical application influence solid-state formulation strategies. Created in BioRender. Wali, A.F. (2026) https://BioRender.com/56k0ply, accessed on 2 February 2026.

The development of solid-state stabilization methods for mRNA therapeutics requires specific approaches because the therapeutic molecules exhibit different properties based on their molecular size, degradation mechanisms and required functional stability levels [11,12,18] (Figure 2).

## 2. Methodology

A systematic literature search was performed to identify relevant peer-reviewed studies addressing ambient stability, dry-state preservation, and solid-state formulation strategies for mRNA therapeutics. Preliminary searches were conducted to determine appropriate Medical Subject Headings (MeSH), keyword variants, and evolving terminology associated with mRNA stabilization, drying technologies, and nanoparticle-based delivery systems. A comprehensive list of keywords and search terms was developed, including messenger RNA stability, mRNA formulation, solid-state mRNA, lyophilized mRNA, freeze-drying of mRNA, spray-dried mRNA, glass-transition temperature (Tg), trehalose stabilization, sucrose stabilization, residual moisture, lipid nanoparticles (LNPs), dry powder vaccines, ambient-stable biologics, mucosal mRNA delivery, oral mRNA delivery systems, and solid-state biologics. Searches were conducted across major scientific databases including PubMed, Scopus, Web of Science, and Embase. Boolean operators and database-specific filters were applied where appropriate to refine relevance. Additionally, reference lists of all eligible articles were manually screened to identify further relevant studies not captured during the initial database search.

## 3. Principles of Dry and Solid-State Stabilization

Building on the modality-dependent stability requirements discussed above, the conversion of mRNA formulations from aqueous suspensions into dry or solid matrices represents the most effective strategy for achieving stability under ambient storage conditions. Conversion of aqueous mRNA formulations into solid matrices is the most effective strategy for achieving ambient stability, as removal of water suppresses hydrolytic degradation and reduces molecular mobility [15]. Scientists use two independent theoretical models which include the water-replacement hypothesis and vitrification theory to study how excipients protect biomolecules during drying processes and storage time.

**Figure 3:**
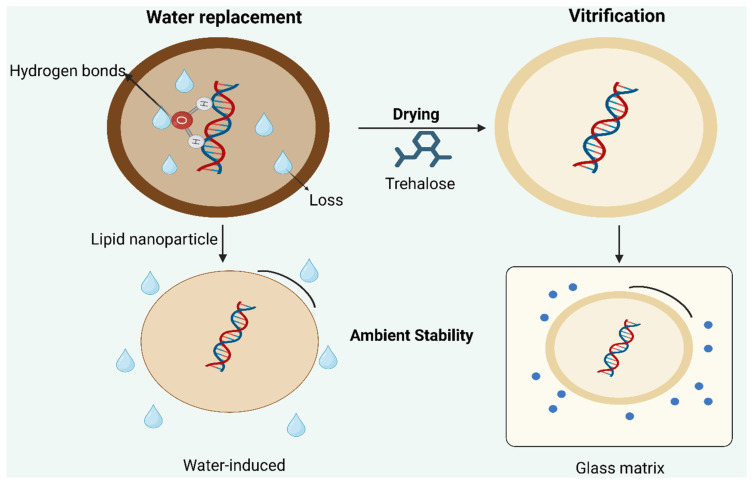
Conceptual diagram of water replacement vs. vitrification. This figure compares two stabilization mechanisms during drying: Hdrogen-bond substitution by sugars (water-replacement hypothesis) and kinetic immobilization within an amorphous glassy matrix (vitrification theory). It highlights how combined effects reduce molecular mobility and degradation rates in solid-state mRNA formulations. Created in BioRender. Wali, A.F. (2026) https://BioRender.com/56k0ply, accessed on 2 February 2026.

The water-replacement hypothesis shows that hydroxyl-rich excipients including trehalose and sucrose create hydrogen bonds with mRNA backbone structures and nucleobases which maintains their original molecular structures when water evaporates [15,16]. In solid-state mRNA formulations, the stabilizer content is typically expressed as % (*w*/*v*) cryo-/lyoprotectant relative to buffer rather than a fixed molar ratio to mRNA, with most successful lyophilized mRNA-LNP vaccines using ~5–10% sucrose or trehalose as the primary excipient; combinations such as 9% trehalose + 1% PVP (*w*/*v*) have been identified to further enhance thermal stability and physical integrity during storage at elevated temperatures. In these systems, the mRNA is encapsulated within lipid nanoparticles (LNPs) rather than existing as a free macromolecule in the solid, and the stabilizers act to vitrify the external matrix and maintain LNP colloidal integrity during freezing and drying [17].The vitrification theory explains how an amorphous glassy matrix forms with elevated glass-transition temperature (Tg) which physically traps mRNA and its carrier system to decrease both chemical and physical decay processes [18,19]. Glass-transition temperatures (Tg) of the lyophilized matrix are critical for solid stability and are strongly excipient-dependent: sucrose matrices show lower Tg (40–50 °C), while trehalose-rich matrices exhibit much higher Tg (90–100 °C); incorporation of Tg-modifying excipients (e.g., PVP, cyclodextrins) can raise Tg further, facilitating storage at temperatures significantly below the Tg to suppress molecular mobility. Factors influencing Tg in these formulations include excipient composition and ratios, residual moisture content (higher moisture lowers Tg), molecular mobility of the amorphous matrix, and freeze-drying (lyophilization) kinetics and cycle design, which determine ice formation and solute concentration profiles during drying—each of which directly impacts the physical stability and shelf life of the formulation [17,18].The mechanistic comparison between hydrogen-bond substitution and kinetic immobilization in glassy matrices is schematically presented in Figure 3.

The stabilization process in practice depends on two main factors that occur within a rigid solid matrix structure: hydrogen-bond substitution and kinetic immobilization [16,19]. The process of freeze-drying (lyophilization) serves as the primary method for creating solid-state mRNA formulations which are used throughout the industry. The process starts with controlled freezing of the formulation before ice sublimation occurs under reduced pressure, and the process ends with unfrozen water extraction during secondary drying. The biological activity of optimized lyophilized mRNA–lipid nanoparticle (LNP) systems stays active during multiple months of storage at 25 °C, according to [20,21]. However, lyophilization is an energy-intensive and time-consuming process that is highly sensitive to freezing rate, chamber pressure, and secondary drying temperature [20]. The product will experience phase separation when freezing occurs under wrong conditions while the excipients will crystallize, leading to permanent damage of the product’s stability [20,22].

The current drying methods face multiple restrictions that alternative drying techniques including spray-drying, spray-freeze-drying, thin-film drying and foam-drying aim to overcome. The spray-drying method produces micron-scale powders at high speed, but it subjects mRNA and LNPs to strong mechanical forces and hot temperatures during the process [23]. The spray-freeze-drying process protects materials from thermal breakdown because it freezes droplets at low temperatures before they undergo sublimation to create powders which have excellent reconstitution properties [24]. The milder thermal conditions of thin-film and foam-drying processes have proven effective for creating solid dosage forms which work well with microneedle patches and oral thin films [25].

In nano spray drying, droplet formation via vibrating mesh technology, laminar heating architecture, and electrostatic particle collection minimizes particle loss and enables efficient recovery of submicron particles while reducing thermal and mechanical stress compared with conventional spray drying. Critical process parameters including spray mesh size, inlet temperature, solid concentration, and drying gas flow rate directly govern particle size distribution, morphology, encapsulation efficiency, and residual moisture, thereby influencing the physical stability and functional preservation of thermolabile biomolecules like mRNA. Compared with classical spray drying, nano spray drying offers improved control over nanoscale particle engineering, higher collection efficiency for submicron fractions, and suitability for sensitive therapeutics such as proteins and mRNA, SiRNA, making it a relevant platform for solid-state stabilization of advanced biopharmaceuticals [26].

Real-time process control systems play a crucial role in achieving successful implementation of solid-state stabilization methods. The implementation of process-analytical technologies (PATs) which track moisture content and temperature and morphological changes during drying operations has become vital for achieving batch consistency and meeting Good Manufacturing Practice (GMP) requirements [27]. The selection of excipients stands as a critical development step which maintains mRNA stability throughout the process of creating solid-state formulations. The scientific community considers trehalose as the standard stabilizer because it has a high glass-transition temperature and strong hydrogen-bonding ability and does not form crystals [15,28]. The combination of sucrose and mannitol with arginine, histidine, dextran, polyvinyl alcohol polymeric matrices, and specific amino acids leads to matrix modification which enhances mechanical strength and rehydration performance [29]. The most stable materials result from drying processes which produce less than 2% residual moisture because water content above this level reduces Tg values and speeds up degradation, but drying beyond this point leads to materials that lose strength and fail to dissolve correctly [30]. The storage temperature needs to stay at least 20 °C lower than the formulation Tg value to achieve long-term product stability throughout its entire shelf life [28,29].

To facilitate structured comparison of the principal drying technologies used for solid-state stabilization of mRNA therapeutics, a consolidated overview of process parameters, stresses, excipient compatibility, stability outcomes, and scalability considerations is provided in Table 1. This table is explicitly referenced here to improve integration within the manuscript text and to guide readers in correlating drying-induced stresses with formulation design requirements and room-temperature stability performance.

The process of rehydration leads to functional recovery which scientists tend to ignore when studying solid-state stabilization methods. The reconstitution process for dried formulations needs to restore their original particle size distribution, encapsulation efficiency, and translational potency. Research studies primarily measure physical and chemical stability, but they do not assess biological recovery because this factor determines how well treatment methods work [24,27]. The storage environment with elevated humidity will lower Tg values, trigger recrystallization, and accelerate degradation processes which requires packaging systems that manage moisture levels [27].

### Critical Insight: From Empirical Drying to Predictive Stabilization Science

Scientists have achieved more stable mRNA through solid-state conversion, but they continue to evaluate new formulations by conducting experimental studies. Research studies evaluate end points through residual moisture content and glass-transition temperature measurements, yet they do not show how these measurement results affect biological performance. The water-replacement and vitrification frameworks provide important theoretical knowledge, but they do not show the entire organizational pattern of mRNA–lipid matrices [15,18]. The solid formulation implementation reveals nanoscale heterogeneity because the formulation contains three distinct sugar, lipid, and water domains which have different viscosity levels and dielectric constants. The material contains different areas which create specific zones that allow chemical breakdown to continue despite the entire material being in a glassy state [27,29].

The development of new stabilization methods uses predictive design methods to solve the current system problems. Research studies demonstrate that amino-acid–based glasses when combined with zwitterionic polymers and deep eutectic solvent (DES) matrices create stronger hydrogen bonds and better reactive species absorption and improved mechanical properties of solid matrices [30]. Scientists can now predict both degradation routes and formulation behavior through the combination of molecular dynamics simulation progress with spectroscopic mapping and kinetic modeling [27,29].

The evaluation of stability requires tests which go beyond standard physicochemical assessments because it needs to assess product behavior during rehydration and its functional recovery after reconstitution. Physical integrity by itself does not protect translational competence from degradation because this competence reveals itself only during biological testing [24]. The development of mRNA medicines for stable use requires scientists to adopt definitions of stability which match biological systems [14].

## 4. Formulation Strategies for Ambient-Stable mRNA

The creation of stable mRNA therapeutics through ambient conditions needs formulation methods which unite solid-state stabilization methods with industrial production techniques, regulatory standards, and delivery preparedness. The achievement of room-temperature stability requires solutions which extend beyond formulation development because it involves multiple system components including excipient choices, process optimization, packaging construction, and environmental testing protocols [38,39].

In the present review, the majority of excipient strategies discussed refer to formulations in which mRNA is encapsulated within lipid nanoparticle (LNP) systems prior to drying. In such systems, excipients must stabilize both the nucleic acid and the supramolecular lipid assembly. In contrast, carrier-free mRNA formulations—although less common in clinical development—require excipients that directly protect RNA secondary structure without the added complexity of colloidal lipid interfaces. This distinction is critical, as excipients may differentially affect RNA integrity, lipid phase behavior, and nanoparticle colloidal stability [31,38].

The process of freeze-drying (lyophilization) stands as the primary method which scientists use to improve mRNA–lipid nanoparticle (LNP) system stability. The controlled sublimation method of lyophilization protects RNA and lipid components through its process of bulk and bound water removal which creates a glassy matrix that safeguards these components from hydrolytic and oxidative degradation [40,41]. Research has shown that disaccharide cryoprotectants including trehalose and sucrose help maintain particle structure and biological activity of particles during storage at 25 °C for multiple months [32,42]. The process of large-scale lyophilization needs exact management of freezing speed, chamber pressure, and drying temperature to stop lipid phase changes and prevent RNA release and excipient crystal formation [34,41]. The manufacturing speed of conventional lyophilization remains restricted because it requires extended processing times and expensive equipment investments [33].

Beyond vitrification and water-replacement mechanisms, sugars such as trehalose and sucrose play an essential role in preserving colloidal stability of mRNA–LNP systems. During freezing and drying, cryo-concentration and ice-crystal formation may induce lipid phase separation, membrane fusion, or aggregation. Disaccharides modulate interparticle interactions by maintaining hydration shell structure, reducing van der Waals-driven aggregation, and preserving ionizable lipid packing density. However, excessive sugar content may alter osmotic balance during rehydration and influence zeta potential, thereby affecting nanoparticle size distribution and encapsulation efficiency [43].

**Figure 4:**
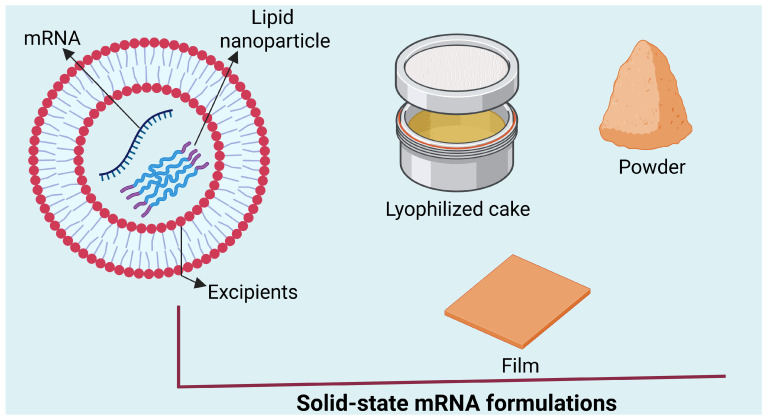
Schematic of solid-state formulations (lyophilized cake, powder, film). Representative examples of lyophilized cakes, spray-dried powders, and thin-film matrices. Each format is annotated with key attributes such as porosity, reconstitution behavior, and compatibility with delivery platforms (e.g., microneedle patches, oral strips). The schematic underscores trade-offs between stability, scalability, and patient-centric design. Created in BioRender. Wali, A.F. (2026) https://BioRender.com/56k0ply, accessed on 2 February 2026.

The current drying methods face multiple limitations which researchers actively work to overcome through new drying approaches. The spray-drying process allows for quick product development through its brief manufacturing period, but it creates harsh conditions which might harm the nanoparticle structure of mRNA–LNPs [36,44]. The spray-freeze-drying process protects materials from heat through its method of freezing small droplets in cold media before they undergo sublimation which produces powders with enhanced reconstitution properties [35,37]. The production of solid dosage forms for microneedle arrays, oral thin films, and non-injectable delivery platforms becomes possible through the use of thin-film drying and foam-drying techniques which work at lower thermal levels [45,46]. The new methods show promise for creating manufacturing systems which can run either continuously or semi-continuously while maintaining their biological properties.The principal solid-state dosage formats derived from these approaches including lyophilized cakes, powders, and films are depicted in Figure 4.

The development of formulation strategies needs to consider all environmental factors which affect stored and distributed products. Laboratory tests which evaluate product stability do not accurately forecast how these products will perform when used in actual field settings, especially in hot tropical environments where temperatures reach above 35 °C and humidity levels exceed 70% [47,48,49,50]. The glass-transition temperature (Tg) of the material decreases when it absorbs even small amounts of moisture during these conditions which also boost molecular movement and speed up degradation reactions [51,52].

The development of packaging design has evolved into an essential element which formulators use to create their formulations, because high-barrier laminates, desiccant systems, and humidity indicators help protect products during transportation and storage [53].

The stability assessment needs to perform mechanical stress testing, vibration simulations, and shipping studies to verify product strength in worldwide distribution networks [54]. The selection of formulation methods depends on financial constraints and environmental standards which need to be met. The process of lyophilization requires significant energy consumption and expensive equipment, but it could reduce costs through three main benefits: removing the need for ultra-cold storage, decreasing product waste, and making distribution operations more straightforward [33,49]. The use of continuous or hybrid drying systems improves both energy efficiency and production capacity but makes it harder to validate and receive regulatory approval for these systems [36,37]. Researchers need to develop environmentally friendly formulations which maintain stability because sustainable manufacturing and packaging practices have become more important [50,51].

The creation of stable mRNA therapeutics which function under typical conditions requires a complete framework combining chemical compound development with production optimization, regulatory approval, and distribution network control [38,39]. The implemented strategies will fulfill their designed objectives to stabilize products and establish mRNA as a therapeutic platform which can be used globally [54].

### Critical Insight: From Empirical Optimization to Predictive Design

Current formulation strategies for ambient-stable mRNA remain largely empirical, relying on iterative trial-and-error approaches to optimize excipient composition, drying parameters, and packaging configurations [55,56]. This fragmented methodology limits mechanistic understanding of RNA–lipid–excipient interactions during drying, storage, and rehydration, thereby constraining rational design.

The next phase of mRNA formulation development will require model-informed and data-driven approaches that integrate molecular simulations, kinetic degradation models, and process-analytical technology (PAT) outputs to predict formulation performance prior to large-scale manufacturing [56,57]. Such predictive frameworks enable simultaneous optimization of stability, manufacturability, and sustainability by linking formulation variables directly to functional outcomes. The convergence of predictive science with scalable processing and environmentally responsible manufacturing will ultimately determine whether ambient-stable mRNA transitions from a promising concept to a globally viable therapeutic reality [50,51,57].

For LNP-encapsulated mRNA systems, optimal trehalose or sucrose concentrations typically range between 10 and 20% *w*/*v* in the pre-lyophilization solution, corresponding to approximately 20–40% *w*/*w* of total solids in the dried matrix. Residual moisture levels should be maintained below 2% to preserve a glass-transition temperature at least 20 °C above intended storage temperature. Amino acids such as histidine are commonly used at 5–20 mM for buffering without excessive Tg depression. Precise optimization remains formulation-specific and requires balancing Tg elevation, lipid compatibility, and reconstitution performance [58].

## 5. Analytical and Stability Testing Paradigms

The development of mRNA therapeutics for clinical use depends on analytical characterization and stability testing to create product quality standards. The unstable nature of mRNA demands a complete analytical system which tracks chemical stability and physical, structural, and functional performance from product development to end-of-life [59,60]. The stability of mRNA–lipid nanoparticle (LNP) formulations depends on three main factors: nucleic acid chemistry, lipid composition, and supramolecular assembly. The analytical framework depends on molecular testing to work with colloidal and biophysical assessments which generate dependable translation results [61,62].

### 5.1. Chemical and Structural Integrity

The RNA backbone and bases undergo degradation because hydrolysis and oxidation reactions affect them [63]. The detection of RNA backbone fragmentation and base modifications uses three main analytical techniques: high-performance liquid chromatography (HPLC), capillary electrophoresis (CE), and mass spectrometry (MS) [64,65]. In stabilized mRNA formulations, these techniques are routinely applied before and after drying and following accelerated storage to quantify full-length RNA retention and identify degradation hot spots. The optimization of HPLC methods which use reverse-phase and ion-pair columns allows for quick measurement of full-length RNA amounts, and LC–MS provides detailed data about oxidation and transesterification reactions [66]. The evaluation of secondary-structure stability under accelerated stress conditions benefits from UV spectroscopy and circular dichroism (CD) analysis, particularly for assessing conformational recovery after rehydration [67].

### 5.2. Lipid and Nanoparticle Characterization

The analytical methods for LNP-based formulations require three main assessments: particle size distribution measurement, zeta potential assessment, and encapsulation efficiency evaluation. The physical stability and morphological consistency of nanoparticles can be evaluated through dynamic light scattering (DLS), nanoparticle tracking analysis (NTA), and cryogenic transmission electron microscopy (cryo-TEM) [68,69]. For dried and reconstituted LNPs, comparative size and morphology analysis before drying, after drying, and post-rehydration provides direct evidence of formulation robustness. The research method uses differential scanning calorimetry (DSC) together with small-angle X-ray scattering (SAXS) to study phase transitions and lipid organization by analyzing their experimental data [70]. The analytical method detects lipid oxidation and phase separation before potency degradation occurs. These techniques are particularly valuable for detecting lipid phase separation or crystallization induced by drying or prolonged storage, which often precede potency loss [71] (Table 2).

### 5.3. Potency and Functional Recovery

The functional assessment of stability known as potency testing connects molecular stability to biological performance. The evaluation of storage-induced potency maintenance needs in vitro translation assays with reporter mRNAs and cell-based assays which measure the amount of expressed protein [72]. In stable mRNA formulations, potency testing is increasingly conducted after rehydration to confirm recovery of translational efficiency rather than solely measuring physicochemical integrity. The evaluation of vaccine potency depends on antigen expression and immunogenicity tests which serve as the primary assessment methods [73]. The current regulatory frameworks lack standardized definitions for nucleic-acid-based product potency [74]. The development of mRNA quality-by-design (QbD) initiatives depends on correlation studies which establish relationships between physical measurements and biological responses. Quality-by-design (QbD) initiatives therefore emphasize correlation studies that link particle size, residual moisture, Tg, and RNA integrity to functional expression outcomes [75,76].

### 5.4. Accelerated and Real-Time Stability Studies

The current ICH stability guidelines which were developed for proteins and small molecules fail to predict the long-term stability prediction of mRNA–LNP products. The storage environment of mRNA–LNP products determines their degradation rate, so researchers need to run accelerated stability tests through Arrhenius-based thermal models and humidity-controlled experiments [77]. Predictive models operating in modern times employ moisture sorption isotherms together with glass-transition behavior to determine product shelf life under various environmental settings [78]. The evaluation of product stability under various conditions needs real-time testing which depends on validated reference materials together with standardized laboratory procedures. For solid-state mRNA formulations, moisture sorption isotherms and Tg-based modeling are increasingly used to define acceptable storage envelopes and packaging requirements [79].

### 5.5. Emerging Analytical Technologies

Scientists will achieve better results in their research of mRNA degradation mechanisms and stabilization processes through the creation of advanced analytical systems. Raman and infrared (IR) spectroscopy enable scientists to perform non-destructive analysis of drying and rehydration processes and have been applied to monitor hydrogen-bond interactions and trehalose vitrification behavior in lyophilized mRNA–LNP matrices [80]. The detection of thermal transitions which show conformational instability becomes possible through differential scanning fluorimetry (DSF) and nano-DSC and has been used to correlate glass-transition temperature shifts with residual moisture–dependent molecular mobility in sugar-stabilized mRNA formulations [81]. Scientists study degradation products at nanoscale levels through single-molecule fluorescence and atomic force microscopy (AFM) including visualization of LNP structural integrity and aggregation following freeze-drying or spray-drying stress [82]. Scientists use machine learning with different assays to generate stability predictions by integrating Tg, moisture content, and particle size distribution with accelerated stability datasets to model degradation kinetics and optimize excipient ratios in mRNA–LNP systems [83,84]. The advanced analytical methods achieve process control through their combined capabilities which provide mechanistic resolution.

### 5.6. Critical Insight: Defining “True Stability”

**Figure 5:**
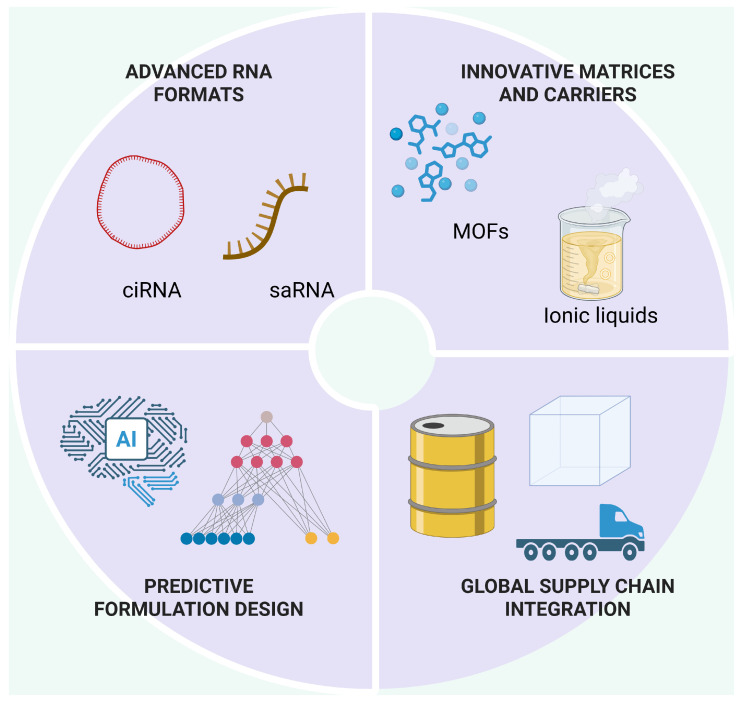
Next-generation stabilization landscape (AI, MOFs, ionic liquids, circular RNA). A roadmap of emerging technologies—including metal–organic frameworks (MOFs), ionic liquids, deep eutectic solvents, and AI-driven predictive design—integrated with advanced RNA modalities (e.g., circular RNA). The figure positions these innovations within a multi-dimensional space of chemical protection, process feasibility, and regulatory readiness. Created in BioRender. Wali, A.F. (2026) https://BioRender.com/56k0ply, accessed on 2 February 2026.

The present emphasis on chemical stability and physical measurements does not reveal how molecular structure relates to biological function. The definition of “true stability” requires translational competence to be preserved because it enables mRNA formulations to produce their designed protein output after storage [85,86]. The goal requires analytical methods which monitor mRNA sequences from their start to their biological end while regulatory organizations create standardized guidelines and predictive models [87]. Emerging stabilization technologies and next-generation predictive platforms that may redefine ambient mRNA stability are summarized in Figure 5.

## 6. Manufacturing, Regulatory and Global Deployment Perspectives

The process of turning laboratory-developed mRNA medicines into worldwide distribution requires more than basic formulation expertise because it needs industrial production capabilities, regulatory standards, and supply chain stability [88]. The production of mRNA therapeutics at commercial scales requires specific manufacturing methods. The enzymatic cell-free mRNA synthesis platform enables fast scale-up, but the following steps need development for reproducible and cost-effective production of mRNA-LNP complexes and their subsequent drying and sterile packaging [89,90].

### 6.1. Manufacturing Challenges

The process of lyophilizing mRNA-LNP combinations through traditional methods requires significant manual work and consumes substantial amounts of power. The development of freeze-drying cycles requires precise planning to achieve uniform product quality throughout thousands of vial production [91]. The transition to large-scale production of mRNA therapeutics through continuous lyophilization processes creates three main problems with ice-front propagation, sublimation dynamics, and residual-moisture control [92]. The process of handling sterile nucleic acids and nanoscale carriers requires strict containment measures and active process surveillance [93]. The combination of production time extensions and cost increases because of complex ambient-stable formulations creates a conflict between product complexity and potential distribution chain elimination benefits [94].

The development of continuous manufacturing systems and modular fill–finish operations serves to solve current production limitations. The combination of continuous freeze-drying platforms with process-analytical technologies (PATs) and model-predictive control systems enables real-time temperature and moisture tracking which leads to better product consistency and shorter production times [95,96]. The combination of robotic systems with closed aseptic processing enhances both biosafety standards and production capacity [97].

### 6.2. Regulatory Considerations

The current regulatory environment shows signs of change, but it remains incomplete. The FDA and EMA now understand mRNA technology as a platform system which enables multiple products to share a common manufacturing process through sequence variations, thus leading to discussions about master comparability protocols [98].

The evaluation process for residual moisture, glass-transition temperature (Tg), and packaging barrier integrity needs clarification for submission purposes.

The existing ICH Q1A (R2) and ICH Q5C documents were created for protein and small molecule products, but they do not fully address the unique chemical-biophysical characteristics of nucleic acid therapeutics [99,100]. Regulatory scientists demand new mRNA guidelines which will integrate stability assessment with potency maintenance and cold-chain independence [101].

The development of analytical methods focuses on three essential quality attributes (CQAs): mRNA integrity, encapsulation efficiency, and translational potency [102].

The lack of standardized definitions and validated stability-potency relationships makes it difficult for sponsors to validate ambient storage periods [103,104]. The World Health Organization (WHO) and Pharmaceutical Inspection Co-operation Scheme (PIC/S) have developed frameworks which link analytical results to functional endpoints for global vaccine distribution standards [105].

### 6.3. Global Deployment and Logistics

**Figure 6:**
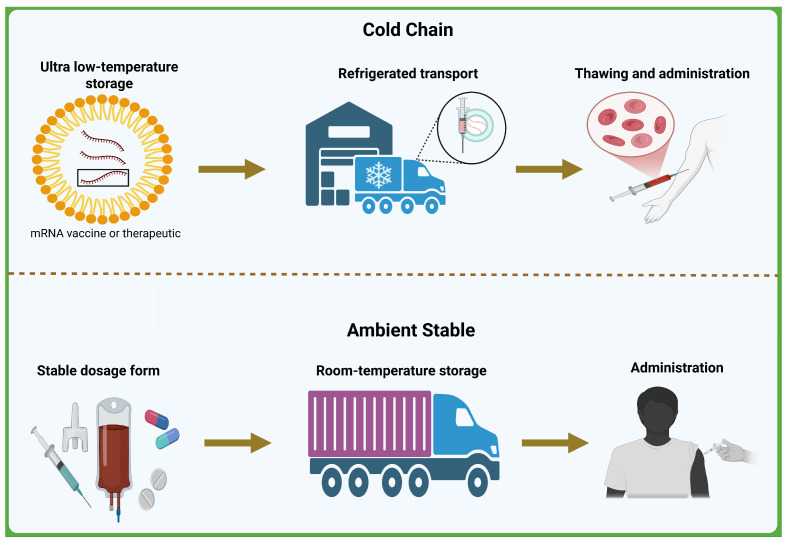
Global deployment and logistics stressors for mRNA therapeutics. A systems-level visualization of environmental and mechanical stress factors encountered during distribution in WHO climatic zones III–IVb. It includes temperature/humidity excursions, vibration profiles, and packaging integrity risks, emphasizing the need for high-barrier laminates and desiccant systems to maintain stability during tropical shipping. Created in BioRender. Wali, A.F. (2026) https://BioRender.com/56k0ply, accessed on 2 February 2026.

The worldwide deployment requirements make ambient stability the most critical factor. The requirement for cold-chain maintenance creates major obstacles for achieving equal healthcare access throughout low- and middle-income countries (LMICs) [106]. The distribution of mRNA vaccines becomes more expensive because of the need for continuous refrigeration, dry ice, and specialized transportation methods which also generate additional greenhouse gas emissions [107]. The elimination of distribution constraints through ambient-stable mRNA formulations enables pharmaceutical supply network utilization for storage under standard pharmacy conditions [108] are summarized in Figure 6.

Products need to withstand tropical environments with temperatures between 30 and 40 °C and humidity levels above 70% during their shipping process [109]. The development of advanced packaging solutions which include multi-layer barrier laminates, desiccant systems, and humidity indicators has evolved into a fundamental aspect of formulation design [110]. The WHO climatic zones III–IVb require regulatory agencies to validate products through tropical stability tests at 30 °C/75% RH for multiple months [111].

Taking deep-cold logistics out of mRNA delivery might cut expenses nearly 60 percent, along with lower carbon output [112]. Better access to healthcare in poorer nations could then support reaching UN’s SDG3 goal for overall health and wellbeing [113].

Obtaining consistent stability rules for nucleic-acid treatments means companies, health regulators, and worldwide medical groups must work together-esting how drugs travel while ensuring factories use smarter processes that mix efficiency without harming the environment [114,115,116].

Obtaining mRNA drugs from lab tests to people everywhere is not just about science-it needs all parts working together. Equity matters, as does access, yet stability in supply also plays a role. This path cannot skip safety or long-term supply chains.

## 7. Outlook and Future Directions—The Road to Shelf-Stable mRNA

What stands out about mRNA treatments lasting at room temperature is how much progress it shows in life medicine research. Achieving real storage stability comes down to three core needsdeep insight into how things work, new methods beyond old practices, and aligned rules across regions.

### 7.1. Mechanistic Insight and Predictive Stability

Scientists need to move away from trial-and-error methods of formulating mRNA therapeutics because they must use molecular design principles to create stable products. Stability engineering requires researchers to merge RNA secondary structure analysis with excipient chemical properties and lipid-matrix interactions through quantitative thermodynamic models. Scientists can predict mRNA degradation mechanisms through precise studies using solid-state NMR, molecular-dynamics simulations, and time-resolved spectroscopy which allow them to forecast hydrolytic and oxidative breakdowns. Scientists can use their gained understanding to choose excipients through scientific methods while conducting cost-effective predictive tests instead of conducting costly trial-and-error experiments [117].

### 7.2. Process Innovation and Manufacturing Scalability

The development of industrial-scale mRNA production requires new manufacturing methods which protect molecular stability while achieving high production rates and consistent results. The development of advanced manufacturing systems includes continuous spray-freeze-drying, thin-film drying, and hybrid modular systems that use real-time process-analytical technologies (PATs). The implementation of digital-twin control systems enables manufacturers to optimize their production lines through automatic parameter adjustments for chamber pressure and residual moisture and glass-transition temperature [118].

### 7.3. Regulatory Harmonization and Global Implementation

The development of standardized definitions for ambient stability requires immediate attention from regulatory bodies. The implementation of standardized definitions which include potency maintenance, humidity resistance, and packaging validation will create better global comparison capabilities and faster approval processes. The transition of sustainability from ethical practice to regulatory requirement has started; manufacturers must now meet three essential criteria for review: carbon-neutral processing, recyclable packaging, and energy-efficient drying [119].

### 7.4. Toward a Resilient, Equitable Future

The development of ambient-stable mRNA medicines will reach one-year storage stability at 25 °C during the upcoming decade through pre-filled vials, microneedle patches, and oral thin-films that need no refrigeration. Orodispersible films (ODFs) are currently considered an exploratory platform for mRNA delivery; however, effective systemic administration requires protection against gastrointestinal degradation and enhancement of epithelial transport. In this context, carrier-based systems, particularly lipid nanoparticles (LNPs) and polymeric nanoparticles incorporating ionizable lipids or mucoadhesive/pH-responsive polymers, are essential to shield mRNA from nuclease activity and promote cellular uptake via endocytosis and transcytosis mechanisms, whereas carrier-free (naked) mRNA formulations are unlikely to achieve therapeutically relevant systemic exposure. The film matrix primarily serves as a secondary stabilization and local delivery scaffold, while nanoparticle encapsulation constitutes the principal protective and absorption-enabling strategy [120,121]. The achievement of this goal requires scientists to develop new manufacturing methods which unite scientific principles with practical manufacturing techniques and worldwide healthcare accessibility. The successful development of mRNA therapeutics will transform these medicines into durable treatments which provide worldwide accessibility and sustainable healthcare solutions for global biopharmaceutical care [122].

**Figure 7:**
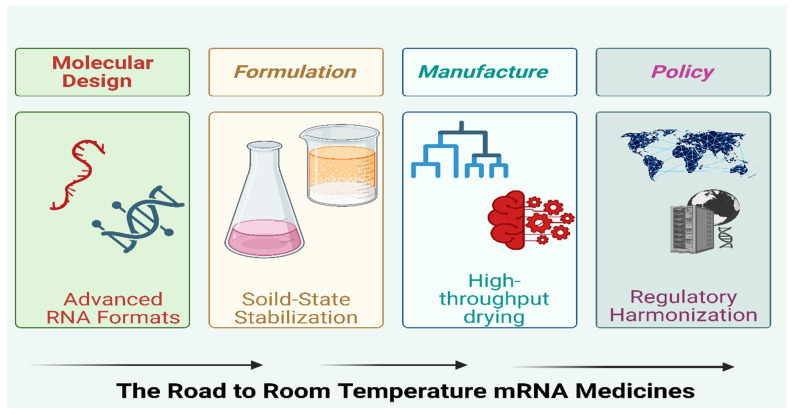
Visionary roadmap linking molecular design → formulation → manufacture → policy. Created in BioRender. Wali, A.F. (2026) https://BioRender.com/56k0ply, accessed on 2 February 2026.

This Figure 7 illustrates the multidisciplinary pillars required to achieve room-temperature storage of mRNA therapeutics. Molecular Design focuses on engineering advanced RNA formats that enhance intrinsic mRNA stability. Formulation strategies such as solid-state stabilization improve durability during storage and transport. Manufacturing innovations, including high-throughput drying technologies, enable scalable production of stable mRNA products. Finally, regulatory harmonization under the Policy domain supports global alignment of standards, facilitating widespread access to next-generation mRNA medicines. Together, these components form the integrated pathway toward achieving room-temperature mRNA therapeutics.

## 8. Conclusions and Key Take-Home Messages

The field of mRNA therapeutics has entered a new phase which focuses on developing stable medications for worldwide distribution without needing cold storage facilities. The achievement of this transformation requires scientists to develop stability as an engineered property which extends across all design levels from molecular structure to lipid components, excipients, drying techniques, and packaging systems. These formulation choices must be guided and validated by fit-for-purpose analytical strategies capable of resolving chemical, physical, and functional stability.

The stability of mRNA therapeutics remains limited because RNA hydrolysis and lipid oxidation and freeze–thaw stress continue to affect aqueous formulations. The most effective method to achieve long-term stability in mRNA formulations involves converting them into solid-state systems through lyophilization, spray-drying, thin-film formation, and novel matrix development. Analytical methods that link residual moisture, glass-transition behavior, particle integrity, and reconstitution performance to biological output will be central to translating these approaches into reliable products. The development of ambient stability for more than 12 months at 25 °C temperatures remains unproven for most mRNA therapeutics outside vaccine applications.

The development of self-amplifying and circular RNA, nano-glass, metal–organic frameworks (MOFs), and AI-based predictive formulation design presents new stabilization methods but faces obstacles related to manufacturing, regulatory approval, and large-scale production. The main obstacles to progress now stem from industrial production challenges, regulatory framework uniformity, and distribution network validation.

The development of ambient-stable mRNA therapeutics requires scientists to work together as a single unit, combining their expertise in chemistry, engineering, regulatory science, and global health. The world will achieve a state where mRNA medicines can be distributed without refrigeration, as they will become accessible, durable, and deployable in all necessary locations.

Cold-chain dependency remains the major bottleneck; solid-state conversion enables ambient stability; mechanistic insight is still incomplete; manufacturing and regulatory alignment are the next frontiers; and achieving room-temperature mRNA therapeutics is now a matter of integration, not invention.

## Figures and Tables

**Table 1:** Comparison of drying techniques and resultant stability metrics.

**Technique**	**Core Steps/Key Parameters**	**Primary Stresses on mRNA/LNP**	**Typical Stabilizers (Examples)**	**Typical Process Ranges**	**Solid-State Output**	**Room-Temp Stability (Illustrative)**	**Scalability/Throughput**	**GMP/PAT Considerations**	**Key Risks/Caveats**	**Therapeutic Application**	**References**
**Lyophilization (freeze-drying)**	Freezing → primary drying (sublimation) → secondary drying; shelf temp, chamber pressure, collapse temp (Tc), product temp (Tp) control	Ice-crystal stress, cryo-concentration, interfacial stress, pH shifts; lipid phase separation; oxidation during storage	Disaccharides: trehalose, sucrose; bulking: mannitol (carefully); amino acids: Arg/His; polymers: dextran, PVA; buffers: histidine	Shelf −40 to −20 °C (freeze); primary −35 to −10 °C/50–200 mTorr; secondary 20–40 °C to <2% residual moisture	Vial “cake” (amorphous glass, occasionally mannitol crystallites)	Months at 25 °C when moisture < 2% and Tg > storage +20 °C	Batch; continuous lyophilization emerging; long cycle times (1–3 days)	PAT: TDLAS, product thermocouples, impedance/DSC mapping; vial-to-vial uniformity	Collapse/melt if Tp > Tc; crystallization of sugars; residual moisture heterogeneity	Lyophilized SARS-CoV-2 mRNA–LNP vaccine, Lyophilized neoantigen mRNA cancer vaccine	[20,21,22,27,28,31,32,33]
**Spray-drying**	Atomization (two-fluid/nozzle) → drying in heated gas → cyclone collection	Shear at nozzle; thermal stress; air–liquid interface; oxidation	Trehalose/sucrose; amino acids; polymers (PVA); antioxidants; leucine (flow aid)	Inlet 60–120 °C; outlet 30–60 °C; solids 1–10% *w*/*v*; residence time <1 s	Micronized amorphous powders	Weeks–months at ≤25–30 °C if Tg high and moisture controlled	Continuous, high throughput; scalable	PAT: outlet temp/humidity; inline particle sizing; solvent handling	Particle fusion at high RH; loss of potency from heat/shear; sticking	Spray-dried inhalable mRNA–LNP dry powder vaccines	[23,34,35]
**Spray-freeze-drying (SFD)**	Atomize into cryogenic liquid (LN2/−80 °C bath) → frozen droplets → vacuum drying	Low thermal load; shear at atomization; cryo-interface stress	Trehalose/sucrose; amino acids; polymers; buffers	Atomization gas 0.5–2 bar; cryogenic bath −80 to −196 °C; primary drying under vacuum	Highly porous, low-density powders (fast rehydration; inhalation-suitable)	Months at 25 °C reported for some systems; moisture sensitivity remains	Semi-continuous; more complex/expensive equipment	PAT similar to lyophilization; cryogen management	Powder friability; moisture uptake; scale-up complexity	Thermostable spray-freeze-dried COVID-19 mRNA vaccine, Porous saRNA–LNP powders	[24,31,36]
**Thin-film/foam-drying**	Spread solution into thin layer; controlled drying under reduced pressure or mild heat (non-frozen)	Interfacial exposure; potential oxidation; minimal freeze stress	Sugars (trehalose/sucrose); polymers; plasticizers (glycerol) for films	Plate 5–30 °C; vacuum; thickness 100–500 µm; moisture target < 2–3%	Uniform amorphous films/foams; patch-compatible	Promising room-temp stability in early studies	Batch/continuous; compatible with patches/oral strips	Need moisture barrier packaging; film uniformity QA	Residual solvent/water gradients; mechanical brittleness	Thin-film freeze-dried mRNA–LNP powders, mRNA-loaded dissolvable microneedle patch	[26,37]

**Table 2:** Comparative matrix of excipients for solid-state stabilization of primarily LNP-encapsulated mRNA formulation (method, excipient, storage T, duration, bioactivity).

**Excipient/Class**	**Primary Stabilization Mechanism(s)**	**Typical Use Level (*w*/*w* of Solids)**	**Glass Transition (Tg)/Crystallization Tendency**	**Compatibility with LNP Lipids**	**Impact on Reconstitution/Potency**	**Caveats/Notes**	**References**
**Trehalose (disaccharide)**	Water replacement; vitrification; H-bonding to phosphate/backbone	20–60%	High Tg (~100–120 °C dry); low crystallization tendency	Generally compatible; preserves bilayer/ionizable lipid packing	Maintains particle size and encapsulation; good potency recovery	Hygroscopic; requires high-barrier packaging to keep RH low	[15,17,28,31,42]
**Sucrose (disaccharide)**	Water replacement; vitrification	20–60%	Tg ~60–80 °C (dry); more prone to collapse vs. trehalose at high RH	Good; widely used in lyophilized biologics	Effective, but lower Tg may limit high-T storage	Crystallization under certain cycles can damage matrix	[15,17,28,41]
**Mannitol (polyol, bulking)**	Cake structure; reduces collapse	5–30%	Crystallizes readily; lowers amorphous fraction if not controlled	Can phase-separate from sugars; affects LNP microenvironment	Improves appearance/handling; may reduce reconstitution if excessive crystals form	Control crystallization (seeding/anneal); avoid dominating the matrix	[29,30,38]
**Arginine/Histidine (amino acids)**	Reduce aggregation; buffer capacity near pH 6–7; interfacial protection	1–10%	No glass; plasticizing potential	Often beneficial for LNP dispersion; histidine common buffer	Faster wetting; can improve translation output post-storage	May depress Tg; ionic interactions can alter zeta potential	[29,32,40]
**Dextran/PVA (polymers)**	Increase matrix strength; moisture control; reduce mobility “hot spots”	1–10%	Raise effective Tg; resist crystallization	Generally compatible; viscosity effects during fill	Enhance cake robustness; slower moisture ingress	Viscosity may hinder atomization; potential impurities (peroxides) in some grades	[29,30]
**Antioxidants (e.g., methionine, tocopherols)**	Scavenge radicals; limit lipid oxidation	0.1–2%	Not glass-formers	Protect ionizable lipids and helper lipids	Preserve potency by curbing oxidative loss	Compatibility/color/assay validation required	[30,47]
**Buffers (histidine, citrate)**	Control pH; minimize base-catalyzed RNA cleavage	2–20 mM (solution prior to drying)	Buffer salts may plasticize; choose volatile vs. non-volatile carefully	Histidine widely used with LNPs	Improves chemical stability during processing and upon rehydration	Beware salt-induced Tg depression; low ionic strength preferred	[62,65,66]

## Data Availability

No new data were created or analyzed in this study.

## References

[1] Das S., Chatterjee A., Manna J., Das Mandal S.K., Maiti T.K., Bhattacharyya T.K. (2025). Systems-integrated thermostable vaccine delivery: Converging cold-chain-free design, AI-augmented formulation, and climate-resilient infrastructure. Mol. Pharm..

[2] Pawar B., Loganathan S., Belliappa K.M., Ranganathan L.B., Thekdi K.P., Hiware S.D. (2026). A comprehensive review of vaccine development: From traditional platforms to messenger RNA (mRNA) technologies. Cureus.

[3] Driskill M.M., Coates I.A., Hurst P.J., Rajesh N.U., Dulay M.T., Waymouth R.M., Akahata W., Matsuda K., Smith J.F., Jacobson G.B. (2025). Lyophilized SARS-CoV-2 self-amplifying RNA vaccines for microneedle array patch delivery. J. Control Release.

[4] Szabó G.T., Mahiny A.J., Vlatkovic I. (2022). COVID-19 mRNA vaccines: Platforms and current developments. Mol. Ther..

[5] Sim E., Kim D., Nguyen T.H., Kim J., Cho H.E., Koo S., Ihm K. (2024). Origin of oxidation variations in ambient-stable β-InSe. ACS Appl. Mater. Interfaces.

[6] Khan M.F., Baudin F., Sudalaiyadum Perumal A., Kamen A.A. (2025). Freeze-drying of mRNA-LNP vaccines: A review. Vaccines.

[7] Parhiz H., Atochina-Vasserman E.N., Weissman D. (2024). mRNA-based therapeutics: Looking beyond COVID-19 vaccines. Lancet.

[8] Huang L., Zhang L., Li W., Li S., Wen J., Li H., Liu Z. (2020). Advances in development of mRNA-based therapeutics. mRNA Vaccines.

[9] Reichmuth A.M., Oberli M.A., Jeklenec A., Langer R., Blankschtein D. (2016). mRNA vaccine delivery using lipid nanoparticles. Ther. Deliv..

[10] Wadhwa A., Aljabbari A., Lokras A., Foged C., Thakur A. (2020). Opportunities and challenges in the delivery of mRNA-based vaccines. Pharmaceutics.

[11] Polack F.P., Thomas S.J., Kitchin N., Absalon J., Gurtman A., Lockhart S., Gruber W.C. (2020). Safety and efficacy of the BNT162b2 mRNA COVID-19 vaccine. N. Engl. J. Med..

[12] Jamous Y.F., Alhomoud D.A. (2023). The safety and effectiveness of mRNA vaccines against SARS-CoV-2. Cureus.

[13] Raut R., Shrestha R., Adhikari A., Fatima A., Naeem M. (2025). Revolutionizing veterinary vaccines: Overcoming cold-chain barriers through thermostable and novel delivery technologies. Appl. Microbiol..

[14] Baindara P., Dinata R., Kumar R. (2026). Yeast-based vaccine platforms: Applications and key insights from the COVID-19 era. Biomolecules.

[15] Kis Z., Kontoravdi C., Shattock R., Shah N. (2021). Resources, production scales and time required for producing RNA vaccines for the global population. npj Vaccines.

[16] Fang E., Liu X., Li M., Zhang Z., Song L., Zhu B., Li Y. (2022). Advances in COVID-19 mRNA vaccine development. Signal Transduct. Target. Ther..

[17] Rahim M.A., Zahran H.A., Jaffar H.M., Ambreen S., Ramadan M.F., Al-Asmari F., Castro-Muñoz R., Zongo E. (2025). Liposomal Encapsulation in Food Systems: A Review of Formulation, Processing, and Applications. Food Sci Nutr..

[18] Wang T., Sung T.-C., Yu T., Lin H.-Y., Chen Y.-H., Zhu Z.-W., Gong J., Pan J., Higuchi A. (2023). Next-generation materials for RNA–lipid nanoparticles: Lyophilization and targeted transfection. J. Mater. Chem. B.

[19] Schoenmaker L., Witzigmann D., Kulkarni J.A., Verbeke R., Kersten G., Jiskoot W., Crommelin D.J. (2021). mRNA-lipid nanoparticle COVID-19 vaccines: Structure and stability. Int. J. Pharm..

[20] Xu J., Wang J., Su X., Qiu G., Zhong Q., Li T., Xia N. (2021). Transferable, easy-to-use and room-temperature-storable PCR mixes for microfluidic molecular diagnostics. Talanta.

[21] Wang D., Li W., Zhang T., Liu X., Jin X., Xu B., Wu J. (2022). Zinc and acetate co-doping for stable carbon-based CsPbIBr2 solar cells with efficiency over 10.6%. ACS Appl. Energy Mater..

[22] Huang Y., Yin T., Wen Y., Qian S., Wu Y., Hao X., Xu L. (2025). Recent advances in CRISPR- and RCA-based biosensing chips and devices for POCT and in situ detection. Moore More.

[23] Alejo T., Toro-Córdova A., Fernández L., Rivero A., Stoian A.M., Pérez L., de Miguel D. (2024). Comprehensive optimization of a freeze-drying process achieving enhanced long-term stability and in vivo performance of lyophilized mRNA-LNPs. Int. J. Mol. Sci..

[24] Gulati G.K., Simpson A.C., MacMillen Z., Krieger K., Sharma S., Erasmus J.H., Khandhar A.P. (2025). Preclinical development of lyophilized self-replicating RNA vaccines for COVID-19 and malaria with improved long-term thermostability. J. Control Release.

[25] Meulewaeter S., Nuytten G., Cheng M.H., De Smedt S.C., Cullis P.R., De Beer T., Verbeke R. (2023). Continuous freeze-drying of messenger RNA lipid nanoparticles enables storage at higher temperatures. J. Control Release.

[26] Jadhav K., Kole E., Abhang A., Rojekar S., Sugandhi V., Verma R.K., Mujumdar A., Naik J. (2025). Revealing the potential of nano spray drying for effective delivery of pharmaceuticals and biologicals. Dry. Technol..

[27] Suzuki Y., Miyazaki T., Muto H., Kubara K., Mukai Y., Watari R., Sato S., Kondo K., Tsukumo S.-I., Yasutomo K. (2022). Design and lyophilization of lipid nanoparticles for mRNA vaccine and its robust immune response in mice and nonhuman primates. Mol. Ther. Nucleic Acids.

[28] Mensink M.A., Frijlink H.W., van der Voort Maarschalk K., Hinrichs W.L. (2017). How sugars protect proteins in the solid state and during drying: Mechanisms of stabilization in relation to stress conditions. Eur. J. Pharm. Biopharm..

[29] Qi S., McAuley W.J., Yang Z., Tipduangta P. (2014). Physical stabilization of low-molecular-weight amorphous drugs in the solid state: A material science approach. Ther. Deliv..

[30] Paczkowska A., Hoffmann K., Andrzejczak A., Pucek W.F., Kopciuch D., Bryl W., Kus K. (2025). The application of mRNA technology for vaccine production—Current state of knowledge. Vaccines.

[31] Li Q., Shi R., Xu H., AboulFotouh K., Sung M.M., Oguin T.H., Weissman D. (2024). Thin-film freeze-drying of an influenza virus hemagglutinin mRNA vaccine in unilamellar lipid nanoparticles with blebs. J. Control. Release.

[32] Pastre A., Brass O., Terreux R., Megy S. (2025). Coarse-grained simulations of RNA-loaded lipid nanoparticles: Implications on RNA stability and antigenic protein expression in mRNA vaccines. J. Phys. Chem. B.

[33] Hsu D., Jayaraman A., Pucci A., Joshi R., Mancini K., Chen H.L., Nachbagauer R. (2025). Safety and immunogenicity of mRNA-based seasonal influenza vaccines in US adults aged 50–75 years. Lancet Infect. Dis..

[34] Wouters O.J., Shadlen K.C., Salcher-Konrad M., Pollard A.J., Larson H.J., Teerawattananon Y., Jit M. (2021). Challenges in ensuring global access to COVID-19 vaccines. Lancet.

[35] Zhang Q., Cao D., Ma Y., Natan A., Aurora P., Zhu H. (2019). Sulfide-based solid-state electrolytes: Synthesis, stability, and potential for all-solid-state batteries. Adv. Mater..

[36] Lu Y., Zhao C.Z., Yuan H., Hu J.K., Huang J.Q., Zhang Q. (2022). Dry electrode technology, the rising star in solid-state battery industrialization. Matter.

[37] Nikodimos Y., Huang C.J., Taklu B.W., Su W.N., Hwang B.J. (2022). Chemical stability of sulfide solid-state electrolytes: Stability toward humid air and compatibility with solvents and binders. Energy Environ. Sci..

[38] Karaman E., Yavuz A., Karakas E., Balcioglu E., Karaca B., Doganay H.N., Yildiz O. (2025). Impact of mRNA and inactivated COVID-19 vaccines on ovarian reserve. Vaccines.

[39] Kornienko I.V., Aramova O.Y., Tishchenko A.A., Rudoy D.V., Chikindas M.L. (2024). RNA stability: A review of the role of structural features and environmental conditions. Molecules.

[40] Arte K.S., Chen M., Patil C.D., Huang Y., Qu L., Zhou Q. (2025). Recent advances in drying and development of solid formulations for stable mRNA and siRNA lipid nanoparticles. J. Pharm. Sci..

[41] Ferreira-da-Silva R., Lobo M.F., Pereira A.M., Morato M., Polónia J.J., Ribeiro-Vaz I. (2025). Network analysis of adverse event patterns following immunization with mRNA COVID-19 vaccines. Front. Med..

[42] Viger-Gravel J., Schantz A., Pinon A.C., Rossini A.J., Schantz S., Emsley L. (2018). Structure of lipid nanoparticles containing siRNA or mRNA by dynamic nuclear polarization-enhanced NMR spectroscopy. J. Phys. Chem. B.

[43] Lou J., Wu Z., Cheng Y., Li M., Liu N., Wang Z., Zhang H. (2025). Recent advances in freeze-drying technologies for mRNA vaccines against infectious diseases. Int. J. Pharm..

[44] Shepherd S.J., Han X., Mukalel A.J., El-Mayta R., Thatte A.S., Wu J., Padilla M.S., Alameh M.-G., Srikumar N., Lee D. (2023). Throughput-scalable manufacturing of SARS-CoV-2 mRNA lipid nanoparticle vaccines. Proc. Natl. Acad. Sci. USA.

[45] Engers D., Teng J., Jimenez-Novoa J., Gent P., Hossack S., Campbell C., Newman A. (2010). A solid-state approach to enable early development compounds: Selection and animal bioavailability studies of an itraconazole amorphous solid dispersion. J. Pharm. Sci..

[46] Abdul-Fattah A.M., Truong-Le V., Yee L., Nguyen L., Kalonia D.S., Cicerone M.T., Pikal M.J. (2007). Drying-induced variations in physico-chemical properties of amorphous pharmaceuticals and their impact on stability (I): Stability of a monoclonal antibody. J. Pharm. Sci..

[47] Ishizuka Y., Ueda K., Okada H., Takeda J., Karashima M., Yazawa K., Moribe K. (2019). Effect of drug–polymer interactions through hypromellose acetate succinate substituents on the physical stability on solid dispersions studied by Fourier-transform infrared and solid-state nuclear magnetic resonance. Mol. Pharm..

[48] Skerritt J.H. (2025). Considerations for mRNA product development, regulation and deployment across the lifecycle. Vaccines.

[49] Pikal M.J. (2004). Mechanisms of protein stabilization during freeze-drying and storage: The relative importance of thermodynamic stabilization and glassy state relaxation dynamics. Drugs Pharm. Sci..

[50] Van L.M., Chen N., Miyazawa K., Zhang M., Landersdorfer C.B., Kirkpatrick C.M., Gao W. (2025). Clinical and quantitative pharmacology considerations of mRNA therapeutics and vaccine development: Bridging translational and platform gaps for enhanced decision making. Clin. Pharmacol. Ther..

[51] Pozdniakova N., Generalov E., Shevelev A., Tarasova O. (2025). RNA therapeutics: Delivery problems and solutions—A review. Pharmaceutics.

[52] Choe J.A., Burger J., Jones J., Panjla A., Murphy W.L. (2025). Opportunities in therapeutic mRNA stabilization: Sequence, structure, adjuvants and vectors. Adv. Ther..

[53] Li X., Li J., Wei J., Du W., Su C., Shen X., Xu M. (2025). Design strategies for novel lipid nanoparticle for mRNA vaccine and therapeutics: Current understandings and future perspectives. MedComm.

[54] Herdiana Y. (2025). Bridging the gap: The role of advanced formulation strategies in the clinical translation of nanoparticle-based drug delivery systems. Int. J. Nanomed..

[55] Tomeh M.A., Smith R.K., Watkinson A. (2025). Recent developments of RNA vaccines and therapeutics: Reagents, formulations, and characterization. Mol. Pharm..

[56] Akingbola A., Adegbesan A., Adewole O., Adegoke K., Benson A.E., Jombo P.A., Aiyenuro A. (2025). The mRNA-1647 vaccine: A promising step toward the prevention of cytomegalovirus infection (CMV). Hum. Vaccin. Immunother..

[57] Seo S.H., Song M.K. (2025). Advancements and challenges in next-generation mRNA vaccine manufacturing systems. Clin. Exp. Vaccine Res..

[58] Liu C., Rcheulishvili N., Shen Z., Papukashvili D., Xie F., Wang Z., Wang X., He Y., Wang P.G. (2022). Development of an LNP-encapsulated mRNA-RBD vaccine against SARS-CoV-2 and its variants. Pharmaceutics.

[59] Farinha S., Mota R., Lopes C., Velez R., Freire M.G., Aguiar-Ricardo A., Lino P.R. (2025). Mitigating shear stress in spray drying for RNA-loaded lipid nanoparticles through process and formulation optimization. AAPS PharmSciTech..

[60] Unruh T., Götz K., Vogel C., Fröhlich E., Scheurer A., Porcar L., Steiniger F. (2024). Mesoscopic structure of lipid nanoparticle formulations for mRNA drug delivery: Comirnaty and drug-free dispersions. ACS Nano.

[61] Munson J., Freeman Stanfield C., Gujral B. (2006). A review of process analytical technology (PAT) in the US pharmaceutical industry. Curr. Pharm. Anal..

[62] Glassey J., Gernaey K., Clemens C., Schulz T.W., Oliveira R., Striedner G., Mandenius C.F. (2011). Process analytical technology (PAT) for biopharmaceuticals. Biotechnol. J..

[63] Vetter F.L., Zobel-Roos S., Mota J.P.B., Nilsson B., Schmidt A., Strube J. (2022). Toward autonomous production of mRNA-therapeutics in the light of advanced process control and traditional control strategies for chromatography. Processes.

[64] Schad M., Gautam S., Grein T.A., Käß F. (2023). Process analytical technologies (PAT) and quality by design (QbD) for bioprocessing of virus-based therapeutics. Bioprocess and Analytics Development for Virus-Based ATMPs.

[65] Kis Z., Tak K., Ibrahim D., Papathanasiou M.M., Chachuat B., Shah N., Kontoravdi C. (2022). Pandemic-response adenoviral vector and RNA vaccine manufacturing. npj Vaccines.

[66] De Vos J., Morreel K., Alvarez P., Vanluchene H., Vankeirsbilck R., Sandra P., Sandra K. (2024). Evaluation of size-exclusion chromatography, multi-angle light scattering detection and mass photometry for the characterization of mRNA. J. Chromatogr. A.

[67] Boman J., Marušič T., Seravalli T.V., Skok J., Pettersson F., Nemec K.Š., Sekirnik R. (2024). Quality by design approach to improve quality and decrease cost of in vitro transcription of mRNA using design of experiments. Biotechnol. Bioeng..

[68] Helgers H., Schmidt A., Lohmann L.J., Vetter F.L., Juckers A., Jensch C., Strube J. (2021). Towards autonomous operation by advanced process control—Process analytical technology for continuous biologics antibody manufacturing. Processes.

[69] Guimaraes G.J., Kim J., Bartlett M.G. (2024). Characterization of mRNA therapeutics. Mass Spectrom. Rev..

[70] Cheng F., Wang Y., Bai Y., Liang Z., Mao Q., Liu D., Xu M. (2023). Research advances on the stability of mRNA vaccines. Viruses.

[71] Elssaig E.H., Ahmed-Abakur E.H., Alnour T.M., Alsubai M.A., Ali A.E., Ullah M.F., Alenzi F.D. (2025). Significant association between genetic polymorphism of IGF2 mRNA binding protein-2 and type 2 diabetes mellitus. J. Clin. Lab. Anal..

[72] Cheneval D., Kastelic T., Fuerst P., Parker C.N. (2010). A review of methods to monitor the modulation of mRNA stability: A novel approach to drug discovery and therapeutic intervention. J. Biomol. Screen..

[73] Whitley J., Zwolinski C., Denis C., Maughan M., Hayles L., Clarke D., Johnson M.R. (2022). Development of mRNA manufacturing for vaccines and therapeutics. Transl. Res..

[74] Camperi J., Chatla K., Freund E., Galan C., Lippold S., Guilbaud A. (2025). Current analytical strategies for mRNA-based therapeutics. Molecules.

[75] Crommelin D.J., Anchordoquy T.J., Volkin D.B., Jiskoot W., Mastrobattista E. (2021). Addressing the cold reality of mRNA vaccine stability. J. Pharm. Sci..

[76] Yu Y.-S., AboulFotouh K., Xu H., Williams G., Suman J., Cano C., Warnken Z.N., Wu K.C.-W., Williams R.O., Cui Z. (2023). Feasibility of intranasal delivery of thin-film freeze-dried, mucoadhesive AS01B-adjuvanted vaccine powders. Int. J. Pharm..

[77] Youssef M., Hitti C., Puppin Chaves Fulber J., Kamen A.A. (2023). Enabling mRNA therapeutics: Current landscape and challenges in manufacturing. Biomolecules.

[78] Hu C., Bai Y., Liu J., Wang Y., He Q., Zhang X., Liang Z. (2024). Research progress on the quality control of mRNA vaccines. Expert Rev. Vaccines.

[79] Ma M., Balasubramanian N., Dodge R., Zhang Y. (2017). Challenges and opportunities in bioanalytical support for gene therapy medicinal product development. Bioanalysis.

[80] Huang D., Li N., Dong X. (2024). Advances in mRNA vaccine research in the field of quality control. Biologicals.

[81] Takada K., Taguchi K., Samura M., Igarashi Y., Okamoto Y., Enoki Y., Matsumoto K. (2025). SARS-CoV-2 mRNA vaccine-related myocarditis and pericarditis: Analysis of the Japanese adverse drug event report database. J. Infect. Chemother..

[82] BenDavid E., Ramezanian S., Lu Y., Rousseau J., Schroeder A., Lavertu M., Tremblay J.P. (2024). Emerging perspectives on prime editor delivery to the brain. Pharmaceuticals.

[83] Wang C., Zhang Y., Dong Y. (2021). Lipid nanoparticle–mRNA formulations for therapeutic applications. Acc. Chem. Res..

[84] Halloy F., Biscans A., Bujold K.E., Debacker A., Hill A.C., Lacroix A., Ghidini A. (2022). Innovative developments and emerging technologies in RNA therapeutics. RNA Biol..

[85] Webb C., Ip S., Bathula N.V., Popova P., Soriano S.K., Ly H.H., Blakney A.K. (2022). Current status and future perspectives on mRNA drug manufacturing. Mol. Pharm..

[86] Vosoughi P., Naghib S.M., Kangarshahi B.M., Mozafari M.R. (2025). A review of RNA nanoparticles for drug, gene and protein delivery in advanced therapies: Current state and future prospects. Int. J. Biol. Macromol..

[87] Knezevic I., Liu M.A., Peden K., Zhou T., Kang H.N. (2021). Development of mRNA vaccines: Scientific and regulatory issues. Vaccines.

[88] Fonseca L., Junior V., Malta-Santos H., Ferreira C., Moret M., Mascarenhas L., Machado B.A.S. (2024). RNA-based vaccine manufacturing: Infrastructure, regulations, and global implications. CLIUM Org..

[89] Skerritt J.H., Tucek-Szabo C., Sutton B., Nolan T. (2024). The platform technology approach to mRNA product development and regulation. Vaccines.

[90] Shukla D., Vora K. (2025). Regulatory and global challenges in the approval, accessibility, and monitoring of mRNA vaccines post COVID-19: A review. J. Regul. Sci..

[91] Patel A., Patel R. (2023). Analytical method development for biologics: Overcoming stability, purity, and quantification challenges. J. Appl. Opt..

[92] Dweh T.J., Wulu G.J.E., Jallah J.K., Miller D.L., Sahoo J.P. (2025). Innovations in RNA therapeutics: A review of recent advances and emerging technologies. Nucleosides Nucleotides Nucleic Acids.

[93] Chakraborty C., Sharma A.R., Sharma G., Lee S.S. (2021). Therapeutic advances of miRNAs: A preclinical and clinical update. J. Adv. Res..

[94] Kis Z., Kontoravdi C., Dey A.K., Shattock R., Shah N. (2020). Rapid development and deployment of high-volume vaccines for pandemic response. J. Adv. Manuf. Process.

[95] Lokras A., Foged C., Thakur A. (2021). Engineering of solid dosage forms of siRNA-loaded lipidoid–polymer hybrid nanoparticles using a quality-by-design approach. Design and Delivery of siRNA Therapeutics.

[96] Luo W.C., Beringhs A.O., Kim R., Zhang W., Patel S.M., Bogner R.H., Lu X. (2021). Impact of formulation on the quality and stability of freeze-dried nanoparticles. Eur. J. Pharm. Biopharm..

[97] Goswami J., Cardona J.F., Hsu D.C., Simorellis A.K., Wilson L., Dhar R., Ward D. (2025). Safety and immunogenicity of mRNA-1345 RSV vaccine coadministered with influenza or COVID-19 vaccine in adults ≥50 years: A phase 3 trial. Lancet Infect. Dis..

[98] Vallan A., Fissore D., Pisano R., Barresi A.A. (2023). Temperature measurements as a process analytical technology for monitoring pharmaceutical freeze-drying. Pharmaceutics.

[99] Davies J.G., Gao D., Kim Y.J., Harris R., Cash P.W., Schofield T.L., Qin Q. (2017). ICH Q5C stability testing of biotechnological/biological products. ICH Quality Guidelines: An Implementation Guide.

[100] McMahon M.E., Abbott A., Babayan Y., Carhart J., Chen C.W., Debie E., Wu Y. (2021). Considerations for updates to ICH Q1 and Q5C stability guidelines. AAPS J..

[101] Ermer J. (2025). ICH Q2 (R2): Validation of analytical procedures. Method Validation in Pharmaceutical Analysis.

[102] Ruppl A., Kiesewetter D., Koell-Weber M., Lemazurier T., Süss R., Allmendinger A. (2025). Formulation screening of lyophilized mRNA-lipid nanoparticles. Int. J. Pharm..

[103] Zimmermann C.M., Deßloch L., Jürgens D.C., Luciani P., Merkel O.M. (2023). Evaluation of the effects of storage conditions on spray-dried siRNA-LNPs before and after subsequent drying. Eur. J. Pharm. Biopharm..

[104] Crommelin D.J., Florence A.T. (2013). Towards more effective advanced drug delivery systems. Int. J. Pharm..

[105] Wu S.Y., McMillan N.A. (2009). Lipidic systems for in vivo siRNA delivery. AAPS J..

[106] Ananworanich J., Lee I.T., Ensz D., Carmona L., Schaefers K., Avanesov A., Paris R. (2025). Safety and immunogenicity of mRNA-1010 seasonal influenza vaccine: Phase 1/2 trial. J. Infect. Dis..

[107] Chalkias S., Dennis P., Petersen D., Radhakrishnan K., Vaughan L., Handforth R., Das R. (2025). Efficacy, immunogenicity, and safety of next-generation mRNA-1283 COVID-19 vaccine (NextCOVE). Lancet Infect. Dis..

[108] Saffie-Siebert S., Torabi-Pour N., Gibson A., Sutera F.M., Dehsorkhi A., Baran-Rachwalska P., Quinn S. (2024). Large-batch manufacturing process for silicon-stabilized lipid nanoparticles. Mol. Ther. Methods Clin. Dev..

[109] Dormenval C., Lokras A., Cano-Garcia G., Wadhwa A., Thanki K., Rose F., Foged C. (2019). Factors of importance for spray drying of siRNA-loaded hybrid nanoparticles for inhalation. Pharm. Res..

[110] Li X., Jin S., Guo S., Yang D., Sai W., Qiu X., Li M. (2024). Platform technology in global vaccine regulation: Development and applications with insights from China. Vaccines.

[111] Meyer R.A., Trabulo S., Douthwaite J.A., Santos J.L. (2022). Roadmap to the development of mRNA therapeutics. Messenger RNA Therapeutics.

[112] Das A., Pathak K., Saikia R., Pathak M.P., Gogoi U., Das D., Sonowal S. (2019). Clinical translation and regulatory aspects of nanocarriers. Nanocarriers for Nucleic Acids and Proteins.

[113] Ouranidis A., Vavilis T., Mandala E., Davidopoulou C., Stamoula E., Markopoulou C.K., Kachrimanis K. (2021). mRNA therapeutic modalities design, formulation and manufacturing under pharma 4.0. Biomedicines.

[114] Makkar S.K. (2025). Advances in RNA-based therapeutics: Breakthroughs and future perspectives. Front Genet..

[115] Niazi S.K. (2022). Making COVID-19 mRNA vaccines accessible: Challenges resolved. Expert Rev. Vaccines.

[116] Saxena S., Mandrah V., Tariq W., Das P., Sambhav K., Devi S.H., Wali S. (2025). The future of mRNA vaccines: Potential beyond COVID-19. Cureus.

[117] Taning C.N., Arpaia S., Christiaens O., Dietz-Pfeilstetter A., Jones H., Mezzetti B., Smagghe G. (2020). RNA-based biocontrol compounds: Current status and perspectives. Pest Manag Sci..

[118] Warda A.K., Tempelaars M.H., Boekhorst J., Abee T., Nierop Groot M.N. (2016). Identification of CdnL involved in repair and outgrowth of heat-damaged *Bacillus cereus* spores. PLoS ONE.

[119] Borah P., Deb P.K., Al-Shar’I N.A., Dahabiyeh L.A., Venugopala K.N., Singh V., Jaradat D.S.M. (2021). Perspectives on RNA vaccine candidates for COVID-19. Front. Mol. Biosci..

[120] Hoffmann E.M., Breitenbach A., Breitkreutz J. (2011). Advances in orodispersible films for drug delivery. Expert Opin. Drug Deliv..

[121] Friis K.P., Gracin S., Oag S., Leijon A., Sand E., Lindberg B., Lázaro-Ibáñez E., Lindqvist J., Whitehead K.A., Bak A. (2023). Spray dried lipid nanoparticle formulations enable intratracheal delivery of mRNA. J. Control. Release.

[122] Ferlak J., Guzenda W., Osmałek T. (2023). Orodispersible films—Current state of the art, limitations, advances and future perspectives. Pharmaceutics.

